# Development of Free Testosterone Chemiluminescence Detection Kit and Its Clinical Application

**DOI:** 10.1155/jamc/5165899

**Published:** 2026-04-28

**Authors:** Shuang Han, Min Liu, Simin Zhao, Wenjun Yan, Zhixian Lu, Xuan Huang

**Affiliations:** ^1^ Department of Pathology, Affiliated Hospital of Jiangnan University, Wuxi, Jiangsu, China, jiangnan.edu.cn; ^2^ Wuxi Medical College, Jiangnan University, Wuxi, Jiangsu, China, jiangnan.edu.cn; ^3^ Department of Laboratory Medicine, Affiliated Hospital of Jiangnan University, Wuxi, Jiangsu, China, jiangnan.edu.cn

**Keywords:** chemiluminescence kit development, free testosterone, hormone markers, performance evaluation

## Abstract

**Background:**

Free testosterone (FT), the bioactive form comprising 1%–2% of total testosterone, directly enters cells. Unlike total testosterone, FT levels are less affected by sex hormone–binding globulin and better reflect biological activity. Accurate serum FT measurement is crucial for diagnosing conditions like male hypogonadism, PCOS, metabolic syndrome, osteoporosis, and Alzheimer’s. However, existing methods suffer from inadequate sensitivity, complexity, and high cost, necessitating improved detection technologies. We developed and evaluated a chemiluminescence assay kit for FT.

**Methods:**

FT was quantified using a competitive immunoassay where sample FT competes with acridinium ester‐labeled testosterone derivatives for binding to biotinylated antitestosterone antibodies on magnetic beads. Key parameters (magnetic bead concentration, biotinylation, labeling, and antibody/derivative concentrations) were optimized. Kit performance was rigorously assessed for linearity, limit of blank (LoB), accuracy, precision, stability, specificity, and clinical relevance. FT levels were measured in 1615 male and 2035 female patient samples to analyze clinical significance.

**Results:**

The assay demonstrated excellent linearity (*r* > 0.99), low LoB (0.021 pg/mL), high accuracy (deviation < 5%), precision (CV < 5%), and 12‐month stability. Specificity testing showed no cross‐reactivity. Method comparison with 392 clinical samples yielded a strong correlation (*r* = 0.9941). Analysis of patient samples revealed significant FT level differences among males with various diagnoses: lower levels in prostate cancer patients and higher levels in conditions like hair loss.

**Conclusion:**

The developed chemiluminescence FT assay kit exhibits superior performance, low cost, and high automation, fully meeting clinical requirements. FT measurement provides a valuable reference for diagnosing and assessing specific diseases, aiding improved clinical management.

## 1. Introduction

Testosterone is the principal male sex hormone (androgen) with the molecular formula C_19_H_28_O_2_. While it is also crucial for women, it is a hormone secreted by the testes of men and the ovaries of women. The adrenal glands also secrete small amounts of testosterone. It plays a role in maintaining muscle strength and mass, bone density, and strength and enhances energy and physical performance. Testosterone was first identified as a secretory glycoprotein in the culture medium of mouse testicular support cells and is a relatively small cytoskeletal‐associated protein that can bind tightly to the cell surface through receptor‐associated proteins [1, 2].

In clinical tests, serum testosterone levels in patients with Type 1 and 2 diabetes are significantly reduced, and serum testosterone levels are negatively correlated with blood glucose concentration and disease duration. Testosterone has a significant protective effect on pancreatic islet cells and promotes insulin synthesis [3]. For male patients, elevated serum testosterone levels are observed in the following diseases: Leydig cell tumors, true precocious puberty, familial incomplete pseudohermaphroditism type II, complete or incomplete testicular feminization, male infertility syndrome, Reifenstein syndrome, and the use of drugs with androgenic effects, such as phenytoin and danazol [4]. Decreased serum testosterone levels is observed in the following diseases: congenital dysgenesis of the seminiferous tubules, anorchism, cryptorchidism, primary hypogonadism due to underdeveloped or nondeveloped interstitial cells, hypogonadism due to low secretion of gonadotropins, isolated LH deficiency syndrome, obesity‐related hypogonadism, hyperprolactinemia, Cushing’s syndrome, congenital adrenal hyperplasia due to deficiency of enzymes, such as 3β‐hydroxysteroid dehydrogenase and 17‐hydroxylase, and the use of drugs that inhibit testosterone synthesis, such as aminoglutethimide, spironolactone, marijuana, ketoconazole, and dichlorodiphenyltrichloroethane [5–10]. In female patients, elevated serum testosterone levels have been observed in some cases of polycystic ovary syndrome (PCOS), theca cell hyperplasia, ovarian masculinizing tumors, and adrenal cortical hyperplasia [11, 12]. After testosterone is secreted into the blood, 98% is bound to sex hormone–binding globulin (SHBG), and a small amount is bound to albumin and cortisol‐binding globulin. Approximately 1%–2% of total testosterone is in the free state. The level of SHBG in the blood almost determines the amount of free testosterone (FT). The ratio of total testosterone to SHBG (T/SHBG) is known as the FT index. Determination of FT can overcome the influence of changes in the concentration of testosterone‐binding proteins and directly reflect the biological activity of hormones. In many women with hypertrichosis or low SHBG levels, serum total testosterone is often within the normal range, and the measured FT level is significantly higher than 90% [13, 14]. FT can reflect the characteristics of some diseases more accurately than total testosterone and has more significance in clinical diagnosis. Measuring FT levels is also a way to indirectly measure SHBG levels in the blood. Measurement of FT can be used to monitor Alzheimer’s disease in older men, and low levels of FT indicate a high risk of Alzheimer’s disease [15, 16].

The clinical detection of FT levels in the serum is crucial for diagnosing related diseases. Accurate quantification of FT remains methodologically challenging, with approaches broadly categorized as indirect (calculation‐based) or direct (physical separation‐based).

### 1.1. Indirect Methods Include

1. Analog immunoassay (AIA), where labeled testosterone analogs compete with endogenous FT. While operationally simple, AIA suffers from low specificity, significant SHBG sensitivity, and poor correlation with reference methods, rendering it clinically unreliable;
2. Equilibrium binding equations (e.g., Vermeulen model), which calculate FT from total testosterone, SHBG, albumin, and dissociation constants. This approach demonstrates algorithmic variability across laboratories and compromised accuracy during hypoalbuminemia.

### 1.2. Direct Methods Comprise

1. Equilibrium dialysis coupled with LC–MS/MS (ED–LC/MS)—the reference method—which separates FT through semipermeable membranes. Despite gold‐standard accuracy, ED–LC/MS requires large sample volumes (≥ 0.5 mL) and prolonged processing (24–48 h) and exhibits high operational complexity;
2. Ultrafiltration–LC/MS (UF–LC/MS) employing centrifugal separation. Though faster than ED, reproducibility is compromised by membrane variability and operational sensitivities;
3. Immunoextraction–LC–MS/MS utilizing antibody‐mediated enrichment. While offering enhanced specificity, this method demands costly instrumentation and faces antibody batch variability [15, 16]. Critical limitations persist that indirect methods lack diagnostic reliability due to inherent inaccuracies, while direct methods face scalability barriers (cost, throughput, and technical demands). This methodological landscape underscores the urgent need for robust alternatives.

Recent guidelines, such as the “Summary of the clinical practice manual for late‐onset hypogonadism” [17], emphasize the clinical importance of measuring FT, particularly in Asian populations where variations in SHBG levels may influence the interpretation of total testosterone. These guidelines support the need for accurate and direct FT measurement methods, reinforcing the rationale for developing the chemiluminescence‐based kit described in this study.

Compared with the current methods for detecting FT, chemiluminescence immunoassays (CLIAs) have the advantages of high sensitivity, good specificity, simple operation, rapidity, strong anti‐interference ability, and low cost, making them suitable for large‐scale clinical detection and research applications. However, there are currently few CLIA kits for detecting FT content, both at home and abroad. Therefore, it is necessary to develop a more sensitive, specific, and safe CLIA for the detection of FT.

## 2. Materials and Methods

### 2.1. Research Population and Ethical Approval

In total, 4,042 patient samples were collected from the Affiliated Hospital of Jiangnan University between March 2023 and February 2024. All samples underwent rigorous screening, and those exhibiting hemolysis or insufficient volume were excluded from further analysis. The research protocol (approval number: LS2024608, Date: February 17, 2024) was approved by the Human Ethics Review Committee of the Affiliated Hospital of Jiangnan University, indicating that we have adhered to ethical standards.

### 2.2. Main Reagents and Materials

FT antigen (catalog no. FT2011), FT antibody (catalog no. FT3001), and testosterone analogs (catalog no.: FT4001) were provided by Zhenjiang Airenake Biotechnology Co., Ltd. (Zhenjiang, China); magnetic bead reagents were purchased from Thermo Fisher Scientific (China), catalog no. 88817. Chemiluminescent and immunoluminescence instruments were purchased from Faponbiotech (Guangzhou, China), Model: Shine i8000. The pH meter was purchased from Mettler Toledo (Switzerland).

### 2.3. Reagent Preparation

Magnetic bead reagent preparation: Initially, 2.8 μm magnetic beads were selected for the process. The beads were washed with 0.1 mol/L hydrochloric acid and subsequently activated by incubation with 10 mmol/L EDC (1‐(3‐dimethylaminopropyl)‐3‐ethylcarbodiimide) and 5 mmol/L NHS (N‐hydroxysuccinimide) in MES buffer (pH 6.0) for 30 min at room temperature to activate under shaking conditions. Next, a streptavidin solution (concentration of 10 μg/mL) was mixed with the magnetic beads and incubated at 37°C for 2 h to coat the streptavidin on the surface of the magnetic beads. Finally, the magnetic beads were suspended in a solution containing 0.1% bovine serum albumin and 0.05 M Tris‐HCl buffer (pH 7.4).

Calibrators: A set of FT solutions of known concentrations was used to construct a standard curve. Calibrator concentrations were 0, 5, 10, 20, 50, 100, 200, and 400 pg/mL. Calibrators were prepared in a human serum matrix to better simulate a clinical sample environment.

Quality control (QC) products: FT was diluted in a human serum matrix to prepare QC products at concentrations of 5 and 150 pg/mL.

Labeled solution: A solution of testosterone analogs labeled with acridinium ester. The concentration of the testosterone analogs in the labeled solution was 1 μmol/L, and the labeling efficiency of acridinium ester was over 90%, ensuring that the labeled substances have high chemiluminescence activity.

Biotin antibody solution: The antibodies specifically bind to testosterone, which is labeled with biotin. The antibody concentration was 5 μg/mL, and the molar ratio of biotin to antibody was 1:5. This solution was prepared in a solution containing 0.1% bovine serum albumin and 0.05 M Tris‐HCl buffer (pH 7.4).

Substrate solution: A specific substrate solution was used to trigger a chemiluminescence signal from the labeled substances. The main components of the substrate solution are hydrogen peroxide and sodium hydroxide, with concentrations of 0.1 and 0.25 mol/L, respectively. When the substrate came into contact with labeled substances, it triggered a strong chemiluminescent reaction.

Washing solution: The magnetic beads were washed, and unbound substances were removed to reduce the impact of nonspecific binding on the test results. The main components of the washing solution were 0.05 M Tris‐HCl buffer (pH 7.4) and 0.1% Tween‐20 surfactant.

### 2.4. Reaction Process

**FIGURE 1:**
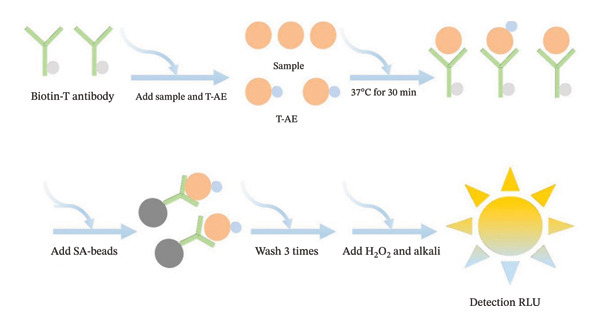
Schematic diagram of the detection process of the free testosterone test kit.

FT content was measured using a competitive method. FT and testosterone derivatives compete for binding to biotinylated testosterone antibodies. The sample was incubated at 37°C for 30 min, then incubated with magnetic bead solution for 5 min. Magnetic separation is achieved by applying a magnetic field. The sample was washed thrice with a washing solution to form an antibody–antigen–acridinium ester complex. The unbound reactants on the magnetic beads were washed away. Hydrogen peroxide and alkaline solutions were added to detect the luminescence. The luminescence value (RLU) was input into the calibration curve to calculate FT concentration (Figure 1).

### 2.5. Data Analysis

Data were analyzed using SPSS 25.0. Normally distributed continuous variables are expressed as mean ± standard deviation (SD), and non‐normally distributed ones are expressed as median. The performance of the method is assessed in accordance with the EP guidelines established by the Clinical and Laboratory Standards Institute (CLSI).

### 2.6. Linearity

The calibrated diluent with antigen served as the high‐concentration sample, and the diluent alone as the low‐concentration sample. High‐concentration samples near the upper linear range limit were serially diluted to various concentrations, including low‐concentration samples near the lower limit. Each concentration was tested in triplicate, and the mean was calculated. The analyte concentration (*x*‐axis) and average test result (*y*‐axis) were fitted by least‐squares regression. The correlation coefficient (*r*) within the linear range was determined, requiring *r* > 0.99.

### 2.7. Limit of Blank (LoB)

The zero‐concentration calibrator, after dilution, was used as the test sample. Twenty measurements were performed to obtain optical signal values. The mean (*M*) and SD were calculated, and *M*‐2SD was determined. A primary equation was derived from two‐point regression fitting using the zero‐concentration reference and the nearest adjacent concentration calibrator’s data. The optical signal value corresponding to *M*‐2SD was substituted.

### 2.8. Accuracy

Samples with high and low concentrations (5 pg/mL and 200 pg/mL) of FT were prepared. The relative deviation (*B*) between the mean value (*M*) and the reference value was calculated using Equation *B* = (*M*−*T*)/*T* × 100% (*T*‐mark value) after three replicate measurements. The reagent was tested on samples at two concentration levels (5 pg/mL and 200 pg/mL), each measured in triplicate.

### 2.9. Imprecision

Prepare three batches of reagents and perform ten replicate detections for high‐value (100 pg/mL) and low‐value control (5.1 pg/mL) items in each batch. Calculate the mean value (*M*) and SD of the results, and subsequently determine the coefficient of variation (CV) using the formula CV(%) = (SD/*M*) × 100%. In this context, CV denotes the coefficient of variation, *M* represents the mean concentration value, and SD signifies the standard deviation.

### 2.10. Thermal Stability Test

The QCs (10.78 pg/mL and 100.87 pg/mL) of the configured FT kit are placed at 4°C and 37°C, respectively, for 1, 3, 6, 10, 20, 25, and 30 days to test the RLU. The experimental results showed that the deviation of the concentration of the QCs was less than 10% when the kit was placed at 37°C for different times compared to storage at 4°C.

### 2.11. Kit‐Specific Test

The specific substance was prepared at the corresponding concentration, and one batch of the FT detection kit was employed to quantify the specific substance and calculate the cross‐reactivity rate. The cross‐reactivity rate (%) was determined using the formula: (free testosterone concentration/specific substance concentration) × 100%.

### 2.12. Clinical Relevance Assessment

To validate the detection efficacy of the kit developed in this study, a clinical performance evaluation was conducted. A total of 392 plasma samples from patients provided by the Affiliated Hospital of Jiangnan University were analyzed using both the self‐developed chemiluminescence‐based FT detection kit and a commercially available kit (Sinbe, Maglumi 2000, Free Testosterone Assay Kit [Chemiluminescence Method], China). The results were compared, with values measured using the self‐developed kit plotted on the *x*‐axis and those from the commercial kit on the *y*‐axis.

## 3. Results

### 3.1. Optimization of the Working Concentration of Magnetic Beads

The magnetic bead concentration was selected based on the optimized antibody concentration. Specifically, magnetic bead concentrations of 15 mg/mL, 10 mg/mL, 7 mg/mL, and 5 mg/mL were evaluated. According to the experimental results, the optimal luminescence effect was achieved at a magnetic bead concentration of 10 mg/mL. Given that the RLU at each detection point reaches its maximum level when the magnetic bead concentration is 10 mg/mL, the concentration of 10 mg/mL was selected for subsequent experimental procedures (Table 1).

**TABLE 1:** Comparison of luminescence values (RLU) in different magnetic bead concentrations of free testosterone kits.

Free testosterone calibrator	Calibration concentration (pg/mL)	Beads concentration 15 mg/mL	Beads concentration 10 mg/mL	Beads concentration 7 mg/mL	Beads concentration 5 mg/mL
S1	0	7328391	7612893	7427162	7213465
S2	5	1324212	1524681	1125361	1231461
S3	10	712361	763752	714535	709869
S4	25	293812	303452	271829	234265
S5	50	112831	152496	113728	109281
S6	100	70121	76843	73212	71231
S7	200	33238	38754	32142	31271
S8	400	10293	19376	14321	12131

### 3.2. Optimization of FT Antibody Working Concentration

Biotin–antibody solution preparation: Initially, antibodies with specific binding affinity for testosterone were biotinylated. During the biotinylation process, the molar ratio of control biotin to antibody was maintained at 1:5. Subsequently, the biotinylated antibody was dissolved in a diluent solution at predetermined concentrations of 3 μg/mL, 5 μg/mL, and 7 μg/mL.

Preparation of marker–antibody diluent: A stock solution was prepared by dissolving 12.05 g of Tris, 10 g of salicylic acid, and 2.0 g of NaN_3_ in 1 L of purified water. The mixture was stirred at room temperature for 1 h. Following this, 0.998 mL of MgCl_2_·6 H_2_O (1 *M*), 1.027 mL of ZnCl_2_ (0.1 *M*), and 1 g of BSA were added to the solution. The pH of the solution was adjusted to 7.0 ± 0.05. The solution was then filtered and stored at 4°C. Biotinylated testosterone analogs were subsequently dissolved in the diluent at concentrations of 0.01 ng/mL, 0.05 ng/mL, and 0.1 ng/mL. Aliquots of 30 μL of each concentration were used to perform the checkerboard titration method. The final working concentrations were set at 5 μg/mL for the biotinylated antibody and 0.05 ng/mL for the labeled testosterone analog (Table 2).

**TABLE 2:** Checkerboard test data.

pg/mL	Biotin‐T antibody 3 µg/mL			Biotin‐T antibody 5 µg/mL			Biotin‐T antibody 7 µg/mL		
	T‐AE 0.01 ng/mL	T‐AE 0.05 ng/mL	T‐AE 0.1 ng/mL	T‐AE 0.01 ng/mL	T‐AE 0.05 ng/mL	T‐AE 0.1 ng/mL	T‐AE 0.01 ng/mL	T‐AE 0.05 ng/mL	T‐AE 0.1 ng/mL
0	3231421	2340123	20441234	50231231	76123123	9785188	20132312	30384736	34256882
25	2002049	1832032	17424123	4012312	3141232	592948	5235829	6374852	5313152
100	873231	741241	6984241	100413	76141	304294	702065	749958	770922
D/A	61.96%	78.29%	85.24%	7.99%	4.13%	6.06%	26.01%	20.98%	15.51%
F/A	43.62%	40.46%	40.08%	0.20%	0.10%	3.11%	3.49%	2.47%	2.25%

### 3.3. Method Performance Evaluation

#### 3.3.1. Linearity

**FIGURE 2:**
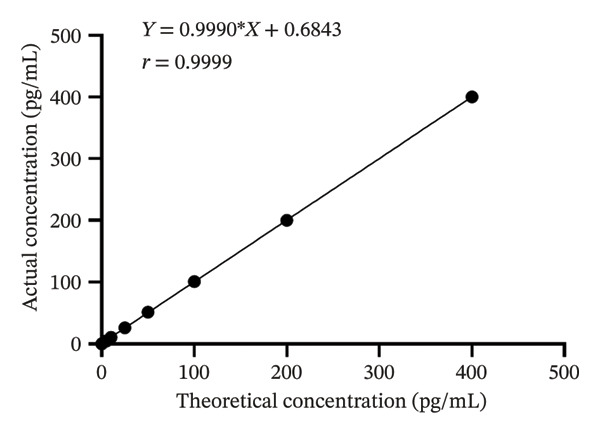
Schematic diagram of the linearity results of the free testosterone detection kit.

Figure 2 presents a linear analysis of FT. The fitted linear regression equation is *Y* = 0.9990X + 0.6843, *r* = 0.9999, demonstrating an excellent linear relationship between the theoretical and actual sample concentrations.

#### 3.3.2. LoB

The results are summarized in Table 3. The concentrations of FT were 0.013, 0.021, and 0.016 pg/mL, and the limit of the FT blank was 0.021 pg/mL.

**TABLE 3:** Verification results of limit of blank of free testosterone.

	**Test 1**	**Test 2**	**Test 3**

M	9898443.05	9230547.10	9779625.65
SD	54762.74	65679.14	161610.73
M‐2SD	9788917.56	9099188.83	9456404.19
LoB (pg/mL)	0.013	0.021	0.016

#### 3.3.3. Accuracy

The experimental results are listed in Table 4. The relative deviation of the FT kit was within 5%, indicating that the kit had good accuracy.

**TABLE 4:** Accuracy test of free testosterone kit.

Concentration (pg/mL)	Test 1	Test 2	Test 3	Mean value (pg/mL)	Relative deviation (%)
5.00	4.98	5.11	4.91	5.01	0.20
200.00	202.10	199.10	201.60	200.35	0.18

#### 3.3.4. Imprecision

The results are summarized in Tables 5 and 6. The intra‐batch and inter‐batch variations of the kit are both less than 3%.

**TABLE 5:** Comparison of intrabatch variation measurement results of free testosterone detection kits.

Number of tests	Low‐value quality control	High‐value quality control
1	5.12	100.38
2	5.29	101.23
3	4.98	100.98
4	5.38	99.98
5	5.02	100.21
6	5.11	100.13
7	5.24	99.87
8	5.12	102.34
9	5.29	100.42
10	4.99	100.71
Fitting concentration *M*	5.15	100.63
Fitting concentration SD	0.14	0.74
Fitting concentration CV	2.70%	0.74%

**TABLE 6:** Comparison of intrabatch variation measurement results of free testosterone detection kits.

Number of tests	Low‐value quality control			High‐value quality control		
	First batch	Second batch	Third party	First batch	Second batch	Third party
1	5.17	5.17	4.85	97.1	103.61	100.2
2	5.16	5	5.13	100.62	102.48	102.03
3	4.9	4.8	4.96	95.29	104.01	99.16
4	4.88	4.82	4.86	96.96	96.92	102.19
5	5.05	4.82	4.97	98.18	103.62	104.92
6	5.18	5.15	4.86	97.96	101.27	95.14
7	5.11	4.8	4.95	104.69	104.85	95.91
8	5.17	4.91	5.07	100.49	103.05	102.38
9	4.8	5.11	4.9	100.37	98.61	102.48
10	5.03	4.98	5.08	99.41	104.91	101.37
Fitting concentration *M*	4.99			100.67		
Fitting concentration SD	0.13			3.01		
Fitting concentration CV	2.67%			2.99%		

#### 3.3.5. Thermal Stability Test

The relative error in the accelerated stability test was maintained within ±5%, demonstrating excellent stability of the kit (Table 7).

**TABLE 7:** Accelerated stability test results of free testosterone kit.

Days	Nominal concentration	Test concentration			Deviation		
		Test 1	Test 2	Test 3	Test 1 (%)	Test 2 (%)	Test 3 (%)
1	10.78	10.89	10.67	10.99	1.02	−1.02	1.95
	100.87	102.23	98.82	101.89	1.35	−2.03	1.01

3	10.78	10.55	10.98	10.59	−2.13	1.86	−1.76
	100.87	101.23	98.89	101.23	0.36	−1.96	0.36

6	10.78	10.45	10.99	10.48	−3.06	1.95	−2.78
	100.87	101.23	100.21	102.89	0.36	−0.65	2.00

10	10.78	10.93	10.69	10.45	1.39	−0.83	−3.06
	100.87	103.23	101.78	98.23	2.34	0.90	−2.62

20	10.78	10.45	10.63	10.99	−3.06	−1.39	1.95
	100.87	99.76	100.98	100.12	−1.10	0.11	−0.74

25	10.78	10.92	10.59	10.87	1.30	−1.76	0.83
	100.87	99.34	96.91	99.23	−1.52	−3.93	−1.63

30	10.78	10.43	10.83	10.69	−3.25	0.46	−0.83
	100.87	99.29	98.91	97.17	−1.57	−1.94	−3.67

FT detection kits were evaluated at 3, 6, 9, and 12 months, respectively. The CV values for QC samples S2, S4, and S6 were consistently below 10%, thereby meeting the specified criteria. These findings indicate that the FT detection kits can be effectively stored at room temperature for up to 12 months. Detailed results are presented in Table 8.

**TABLE 8:** Real‐time stability evaluation results of the free testosterone kit.

Test time (month)	Imprecision (CV%)		
	S2 (%)	S4 (%)	S6 (%)
3	2.91	2.17	5.46
6	2.67	4.95	3.73
9	4.52	2.46	4.21
12	3.61	1.35	4.68

#### 3.3.6. Kit‐Specific Test

The specificity was found to be less than 0.1%, thereby meeting the specified requirements. The results are shown in Table 9.

**TABLE 9:** Specific test results of free testosterone kit.

Cross reactant	Experimental concentration (pg/mL)	Determination of concentration (pg/mL)	Cross‐reactivity rate (%)
DHEA sulfate	5000	0.13	0.0026
Estradiol	1000	0.29	0.029
Cortisol	1000	0.07	0.007
Progesterone	5000	0.25	0.005
Prednisone	1000	0.11	0.011
Corticosterone	1000	0.08	0.008

#### 3.3.7. Clinical Relevance Assessment

**FIGURE 3:**
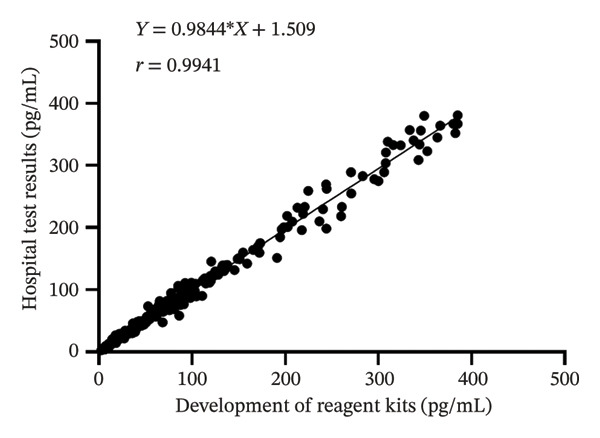
Correlation result diagram of the test kit for free testosterone.

Linear regression analysis revealed a strong linear correlation (*r* = 0.9941), as shown in Figure 3. These findings confirm that the newly developed kit meets clinical detection requirements.

**FIGURE 4:**
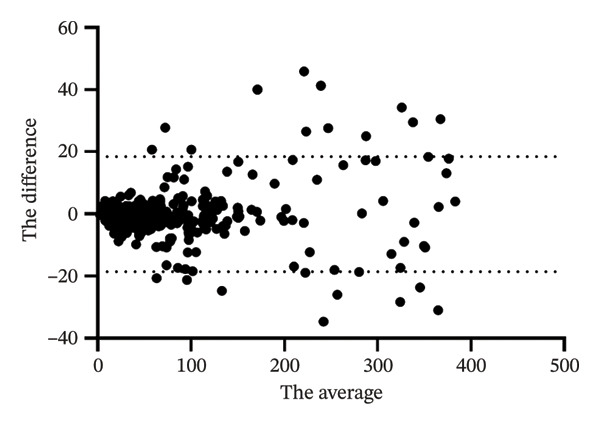
The mean of the measured values of the two methods—the difference between the measured values of the two methods (deviation: −0.03668; SD: 9.452; 95% limits of agreement were −18.56 and 18.49 pg/mL).

We performed a Bland–Altman analysis of the data, and the mean difference between the two measurements was −0.03668 pg/mL, indicating that the kit measurements were very close to the hospital test results with little systematic bias. The 95% limit of agreement was calculated to be −18.56 to 18.49 pg per milliliter (Figure 4). This indicates that most of the sample measurement differences were within this range.

In summary, after many repeated tests, the detection range of the FT chemiluminescence detection kit was 0–400 pg/mL. The limit of the blank was 0.021 pg/mL, and the linear test *r* was 0.9999, which is better accuracy and stability than the same type of kit on the market. By comparing multiple markers, a cheaper acridone ester was selected to ensure labeling efficiency and reduce the production cost, thus reducing the financial pressure on patients when using the FT chemiluminescence detection kit developed in this study.

**FIGURE 5:**
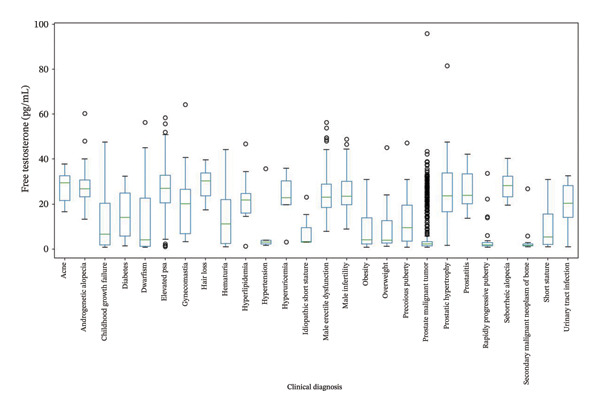
Analysis of the relationship between male free testosterone levels and clinical diagnosis.

**FIGURE 6:**
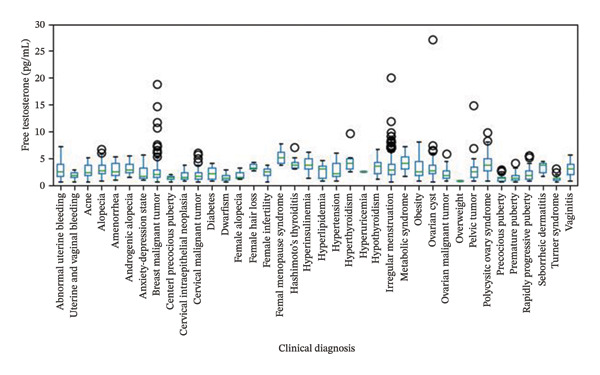
Analysis of the relationship between female free testosterone levels and clinical diagnosis.

We tested 1615 male and 2035 female patient samples using the FT chemiluminescence kit (Figures 5 and 6). Among the males, the FT levels of most patients with prostate malignancy were relatively low, with a median of 2.24 pg/mL and a relatively narrow interquartile range (IQR of 1.82 pg/mL), indicating that the testosterone levels of patients in this diagnostic category were generally low and concentrated. However, there were some substantially high outlier values, suggesting that individual patients may have significantly different pathological features. Patients with diagnoses related to hair loss (such as androgenetic and seborrheic alopecia) generally had higher testosterone levels, with a median of approximately 28 pg/mL and a relatively wide IQR, especially for androgenetic alopecia, with an IQR of 7.56 pg/mL, indicating a large difference in testosterone levels among patients. The maximum values of these diagnostic categories were relatively high, further supporting the association between high testosterone levels and hair loss. The median was lower for diagnoses related to precocious puberty and rapid progression of puberty, but the IQR was relatively wide, especially for precocious puberty with an IQR of 16.03 pg/mL, indicating a large difference in testosterone levels among patients. Some patients had extremely high values, suggesting that these diagnoses may be related to early or rapid changes in sex hormone levels. The median testosterone level was higher in patients with benign prostatic hyperplasia (23.77 pg/mL), and the IQR was relatively wide (17.36 pg/mL), indicating a large difference in testosterone levels among patients. The maximum value was 81.41 pg/mL, indicating that testosterone levels in some patients were significantly elevated, possibly related to the severity of the disease or other factors.

Patients in the different clinical diagnostic categories showed significant differences in FT levels. Some diagnoses (such as prostate malignancy) were associated with lower testosterone levels, whereas others (such as diagnoses related to hair loss) were associated with higher testosterone levels. These results suggest that FT can be an important reference indicator for the diagnosis and assessment of certain diseases.

FT levels in female patients with depression vary significantly. Some patients have significantly elevated hormone levels, which may suggest a significant role of hormone levels in this disease. The FT levels of patients with depression were significantly different, and some patients had higher hormone levels, which may be related to disease severity.

Patients with PCOS typically exhibit abnormal hormone levels. High FT levels may be a characteristic feature of this disease. Hormone levels in patients with precocious puberty are usually higher, which may also be an important sign of the disease. FT levels may become important biomarkers for the better diagnosis and treatment of diseases, such as depression, PCOS, and precocious puberty.

## 4. Discussion

Recent studies have investigated the association between FT and cognitive function in men, particularly among elderly populations. Testosterone may exert neuroprotective effects through mechanisms such as enhancing synaptic transmission, preventing neuronal cell death, and promoting synaptic plasticity. These biological processes may play a critical role in maintaining cognitive performance and delaying the onset of cognitive decline [18]. The research team from Huashan Hospital, affiliated with Fudan University, found that elderly men with low levels of FT exhibit a significantly higher risk of cognitive decline (risk ratio increased by 429%, *p* < 0.001) [15], indicating that FT may serve as a potential biomarker for cognitive decline in this population.

The assay kit developed in this study exhibited a high degree of correlation (*r* = 0.9941; Bland–Altman analysis: mean difference −0.03668 pg/mL, 95% limits of agreement −18.56 to 18.49 pg/mL) with a commercially available kit based on the analysis of 392 clinical samples. The key clinical findings are summarized as follows:

1. Among male patients: The FT level in patients with prostate malignancy was significantly lower compared to other groups (median 2.24 pg/mL vs. overall median 23.77 pg/mL, Mann–Whitney *U* test *p* < 0.001), with a narrow distribution (IQR = 1.82 pg/mL). In contrast, FT levels in patients with hair loss‐related conditions (e.g., androgenetic alopecia) were markedly elevated (median 28 pg/mL, IQR = 7.56 pg/mL; Kruskal–Wallis test *p* = 0.002). In addition, the reference intervals observed in our male cohort were consistent with those reported in healthy Asian populations. For instance, Tsujimura et al. reported serum testosterone levels in healthy Japanese male volunteers using radioimmunoassay, and our findings align with the lower range of FT values observed in their cohort [19]. This concordance supports the clinical applicability of our kit in Asian populations and highlights its potential for standardized use across diverse demographic groups.
2. Among female patients: Patients diagnosed with PCOS or precocious puberty exhibited significantly higher FT levels than those in the healthy control group (median FT level in the PCOS group was 48% higher than that of controls, *p* < 0.01). In comparison, patients with depression displayed high heterogeneity in FT levels, with some individuals showing abnormally elevated values, potentially associated with disease severity.

The detection of FT levels has significant clinical implications. FT is the biologically active form of testosterone in the body and directly reflects the effects of androgens. In men, a decline in FT levels may lead to decreased libido, sexual dysfunction, reduced sperm production, and other issues affecting fertility [20–22]. In women, abnormally elevated FT levels may be associated with PCOS, which presents with symptoms, such as irregular menstruation, hirsutism, and acne. FT is an important indicator of hyperandrogenism [23, 24]. For example, FT levels are significantly elevated in patients with PCOS, and the free androgen index is considered more diagnostically valuable than total testosterone. Additionally, changes in FT levels are associated with metabolic disorders, such as thyroid dysfunction, obesity, and insulin resistance. As men age, FT levels gradually decline, which may be related to symptoms, such as osteoporosis, muscle loss, and fatigue. The detection of FT helps assess the risk of age‐related diseases and guides testosterone replacement therapy. Changes in FT levels may indicate the presence of other diseases. For example, adrenal cortical and testicular tumors may cause elevated FT levels, whereas hypogonadism and liver dysfunction may lead to decreased FT levels. FT testing is not only used for diagnosis but also for monitoring treatment effects. For example, in patients with PCOS, reducing FT levels through treatment can improve symptoms and reproductive outcomes. In men, FT levels can be measured after testosterone replacement therapy to evaluate treatment effects [25, 26]. In summary, FT testing is an important means of assessing androgen levels and related diseases and provides more accurate biological information than total testosterone.

Numerous studies have demonstrated that low testosterone levels in male patients with sepsis are associated with higher mortality rates, longer hospital stays, and poorer clinical outcomes. This may be related to the weakened protective effects of testosterone on immunity, metabolism, and muscle function [27]. Some studies have proposed that testosterone levels may serve as prognostic biomarkers in male patients with sepsis, with lower testosterone levels indicating a higher risk of death. Patients with sepsis often experience significant changes in sex hormone levels, with a notable decrease in testosterone levels in male patients. This decline may be associated with the systemic inflammatory response, stress state, and hypothalamic–pituitary–gonadal axis dysfunction caused by sepsis. Unlike males, estrogen levels in female patients with sepsis are relatively stable or even elevated, which may be related to the lower mortality rate in females with sepsis [28, 29]. In our study, we found that testosterone levels in patients with malignant tumors were relatively low, and low testosterone levels were associated with poor prognosis. Future studies should explore the mechanism of action of testosterone in sepsis and its potential therapeutic value.

The developed kit uses a CLIA and is highly sensitive and capable of detecting FT at the picogram level. Its wide detection and linear ranges cover multiple orders of magnitude to ensure the accuracy of the detection results. This kit specifically recognizes FT and reduces nonspecific binding by optimizing the selection of antibodies and chemiluminescent markers. The operation process is simple, suitable for fully automated operation, reduces manual errors, and improves detection efficiency. This design is advantageous for clinical testing. Compared with traditional methods, the chemiluminescence method can effectively avoid matrix interference and ensure the accuracy of quantitative results. This is particularly important for detecting low‐concentration samples. The preparation method of the kit is simple, low‐cost, and reliable, and the cost is only one‐10th that of mass spectrometry, making it cost‐effective for large‐scale clinical detection.

## 5. Conclusion

This study successfully developed a highly sensitive and cost‐effective CLIA kit for the automated quantification of serum FT. The assay exhibits excellent analytical performance, with a LoB of 0.021 pg/mL, strong linearity (*r* = 0.9999) across the clinically relevant range (0–400 pg/mL), and high precision (CV < 5%) and accuracy (relative deviation < 5%), meeting the requirements outlined in CLSI EP guidelines. Its simplified, fully automatable protocol significantly reduces both turnaround time and manual handling compared to reference methods such as equilibrium dialysis coupled with liquid chromatography–tandem mass spectrometry, while production costs are approximately one‐tenth of those associated with mass spectrometry‐based approaches.

Clinically validated using a large cohort (*n* = 3650), the assay identified significant associations between FT levels and specific pathologies: notably lower median FT concentrations in patients with prostate malignancy (2.24 pg/mL, *p* < 0.001) and elevated levels in individuals with androgenetic alopecia (28.00 pg/mL, *p* < 0.01). This robust assay overcomes key limitations of current FT detection methods—replacing the labor‐intensive nature and high cost of physical separation techniques and the lack of accuracy in indirect immunoassays—thus offering a reliable and scalable solution for diagnosing and managing androgen‐related disorders, including PCOS, precocious puberty, and depressive disorders.

**Nomenclature**
CV: Coefficient of variation
QC: Quality control
PCOS: Polycystic ovary syndrome
SHBG: Sex hormone‐binding globulin

## Author Contributions

Shuang Han was responsible for the study design, writing, and revision. Simin Zhao and Wenjun Yan collected samples. Min Liu was involved in the sample testing and data analysis. Zhixian Lu analyzed the relationship between clinical diagnosis and free testosterone levels. Xuan Huang provided guidance on the overall structure of the study. Shuang Han and Min Liu are the co‐first authors.

## Funding

This work was supported by the Wuxi Institute of Translational Medicine (YJZ202304) and Wuxi Municipal Science and Technology Bureau, “Taihu Light” Technology Research Project (Medical and Health Technology Research Project) (Y20242201).

## Disclosure

All the authors have read and approved the final version of this manuscript. A preprint has previously been published [30].

## Ethics Statement

All procedures were performed in accordance with the ethical standards of the institutional and/or national research committees and the Declaration of Helsinki. This study was approved by the Medical Ethics Committee of the Affiliated Hospital of Jiangnan University (Wuxi, Jiangsu, China) (no. LS2024332), and written informed consent was obtained from all participants before enrollment in the study.

## Consent

Please see Ethics Statement.

## Conflicts of Interest

The authors declare no conflicts of interest.

## Supporting Information

Additional supporting information can be found online in the Supporting Information section.

## Supporting information


**Supporting Information 1** Supporting 1. Date—raw data for assay optimization (magnetic bead concentration, checkerboard titration), linearity, LoB, accuracy, precision, stability, specificity, and method comparison.

**Supporting Information 2** Supporting 2. Free testosterone clinical data—clinical measurements of free testosterone from 3650 patients (1615 male and 2035 female) with corresponding diagnoses for clinical validation.

## Data Availability

The data that support the findings of this study are available in the supporting information of this article.
